# Metolazone and Alzheimer’s Disease Related Dementia (ADRD): Artificial Intelligence Approach To Drug Repurposing

**DOI:** 10.1093/geroni/igaf122.4265

**Published:** 2025-12-31

**Authors:** Ali Ahmed, Sijian Zhang, Yijun Shao, Andrew Zullo, Mo-Kyung Sin, Charles Faselis, Gregg Fonarow, Qing Zeng-Trietler

**Affiliations:** Washington DC VA Medical Center, Washington, District of Columbia, United States; Veterans Affairs Medical Center, Washington, DC, Washington, District of Columbia, United States; George Washington University, Washington, DC, Washington, District of Columbia, United States; Brown University, Providence, Rhode Island, United States; Seattle University, Seattle, Washington, United States; Veterans Affairs Medical Center, Washington, DC, Washington, District of Columbia, United States; UCAL, Los Angeles, California, United States; Veterans Affairs Medical Center, Washington, DC, Washington, District of Columbia, United States

## Abstract

Currently, there is no drug approved for ADRD prevention. Developing new drugs is costly and time-consuming, and drug repurposing is rare and often serendipitous. In our NIA-funded R01 study, we used a Medication-Wide Association Study (MWAS) Plus approach, where “MWAS”, similar to GWAS, identifies medications linked to ADRD from VA’s national pharmacy data using deep learning, and the “Plus” examines these links in propensity score (PS) matched cohorts. Metolazone, a diuretic used in heart failure (HF), emerged at the top of the AI-generated list. To avoid confounding by indication bias, we examined this association in 648,552 HF patients in VA (2000–2017) without baseline ADRD and metolazone use, of whom 5068 were initiated on metolazone. We assembled a PS-matched cohort of 5068 pairs of patients balanced on 65 baseline characteristics and followed them until July 31, 2025. HR (95% CI) for ADRD associated with metolazone was 0.94 (0.83–1.06) and for death was 1.40 (1.35–1.46). To assess the impact of competing risk of death, we compared the median (IQR) time-to-death, which was 5.5 (2.1–9.5) years for non-metolazone and 4.7 (1.3–7.3) years for metolazone. When we examined the association between metolazone and incident ADRD in 9151 (90.3%) patients who died (thus, eliminating the competing risk of death), new ADRD occurred in 12.7% and 9.0% of patients in non-metolazone and metolazone groups, respectively (HR associated with metolazone, 0.86; 95% CI,0.76–0.98). These hypothesis-generating findings need to be replicated and confirmed in clinical trials. These findings also support future use of AI in efficient drug repurposing.

